# Clinical features, effectiveness of therapy and quality of life of patients with type 2 diabetes and comorbid schizophrenia

**DOI:** 10.1192/j.eurpsy.2021.1276

**Published:** 2021-08-13

**Authors:** M. Artemieva, T. Varnakova, I. Danilin, A. Lazukova, R. Suleimanov, V. Sokolov

**Affiliations:** 1 Psychiatry And Medical Psychology, RUDN University, Moscow, Russian Federation; 2 Psychiatry, Psychiatry, Moscow, Russian Federation

**Keywords:** Type 2 Diabetes, schizophrénia, quality of life

## Abstract

**Introduction:**

According to previous studies, about 8,8-14,5% cases of schizophrenia is comorbid to type 2 diabetes. The focus of the study was the evaluation and dynamics of positive and negative symptoms in case of combination of the diseases.

**Objectives:**

100 patients were divided in two groups: 48 patients was assigned to receive a monotherapy treatment with antipsychotic; 52 patients received the combination of antipsychotics, nootropics and antioxidants. The efficiency criterion was the dynamics of the questionnaire The quality of life of patients SF- 36, Hamilton’s scale of Depression and anxiety, overall score on a scale for evaluation positive and negative symptoms (PANSS).

**Methods:**

After treatment the physical component of health is 41,38% in the first group and 56,34% in the second group (p≤ 0,05). The psychical component of health is 39,79% in the first group and 50,8% in the second group (p≤ 0,05). Also statistically confirmed (p≤ 0,05) in the patients of the second group the improvement on the Hamilton’s scale of Depression and anxiety questionnaire and PANSS.

**Results:**

After treatment the physical component of health is 41,38% in the first group and 56,34% in the second group (p≤ 0,05). The psychical component of health is 39,79% in the first group and 50,8% in the second group (p≤ 0,05). Also statistically confirmed (p≤ 0,05) in the patients of the second group the improvement on the Hamilton’s scale of Depression and anxiety questionnaire and PANSS.

**Conclusions:**

According to Quality of Life questionnaire combination of antipsychotic, nootropic, antioxidant is significant more effective than treatment only with antipsychotic.

